# Keratinizing Squamous Cell Carcinoma Masquerading as Basal Cell Carcinoma Constituting a Diagnostic Pitfall: A Case Report With Etiopathogenetic Discourse and Mohs Micrographic Surgical Management

**DOI:** 10.7759/cureus.104371

**Published:** 2026-02-27

**Authors:** Sanjoy Sanyal, Rachel Andrew, Delia Graham-Durand, Suhas Kotbagi, Bernadette Austrie

**Affiliations:** 1 Surgery, All Saints University School of Medicine, Roseau, DMA; 2 Cardiology, Dominica China Friendship Hospital, Roseau, DMA; 3 Cardiology, All Saints University School of Medicine, Roseau, DMA; 4 Behavioral Health, Dominica China Friendship Hospital, Roseau, DMA; 5 Behavioral Health, All Saints University School of Medicine, Roseau, DMA; 6 Internal Medicine, All Saints University School of Medicine, Roseau, DMA; 7 Medical Education and Simulation, All Saints University School of Medicine, Roseau, DMA

**Keywords:** actinic keratosis, alginate wound dressing, basal cell carcinoma (bcc), fitzpatrick skin types, full thickness skin graft, mohs micrographic surgery (mms), non-melanoma skin cancers, pincushioning of graft, squamous cell carcinoma (scc), ultraviolet radiation

## Abstract

Among non-melanoma skin cancers of the face, basal cell carcinomas (BCC) and squamous cell carcinomas (SCC) are the most common. They are close differentials of each other and often constitute diagnostic pitfalls during assessment. This is a case report of the diagnosis and Mohs surgical management of an SCC that was initially clinically diagnosed as BCC. Mohs micrographic surgery (MMS) for cancers of cosmetically sensitive areas such as the face has proved to be a very useful procedure for ensuring 100% tumor removal with maximum normal tissue-sparing. This case report also analyzes the etiological, environmental, and demographic factors that lead to skin cancers of the face, with emphasis on the relationship of Fitzpatrick skin types and ultraviolet (UV) solar radiation vis-à-vis their propensity to cause skin cancers. High index of suspicion followed by early intervention still remains the hallmark of early, accurate diagnosis of skin cancers of the face, with the aim of ensuring high cure rates and minimum facial disfigurements. A case series of this type with longer follow-ups will alleviate the limitations of this case report.

## Introduction

Ever since squamous cell carcinoma (SCC) and basal cell carcinoma (BCC) of skin were described more than a century ago, they have remained the most common non-melanoma skin cancers worldwide. They are close differential diagnoses of each other and still constitute a diagnostic pitfall for clinicians managing such cases [1,2]. BCC arises from the stratum basale of the epidermis, while SCC arises from the prickle cell layer of the epidermis. BCC is the most common skin cancer, with SCC being a close second. SCC typically occurs in older individuals than BCC, with an average age difference of seven years. BCC tends to be locally invasive, while SCC has a propensity toward loco-regional metastases [3-5]. Even though lots of epidemiologic and demographic studies have postulated relationships between Fitzpatrick skin types, ultraviolet (UV) radiation, and skin cancers, when it comes to clinically diagnosing the cancer type in an individual patient, it continues to stump the clinician with surprises [5-8].

We described the case of a younger Afro-Caribbean female patient with a pigmented lesion on the nose that appeared to fit the clinical diagnosis of BCC. Yet, two histopathological examinations (HPE) of biopsy specimens from the lesion revealed it to be SCC. Our case report describes excision of the lesion by Mohs micrographic surgery (MMS), followed by secondary full-thickness skin grafting (FTSG) of the resulting defect. Mohs surgery is based on the technique originally described by Frederick E. Mohs more than a century ago. It revolves around the dual fundamental principles of ensuring 100% tumor removal with maximum normal tissue-sparing. Therefore, it is ideally suited for cancers of cosmetically sensitive areas like the face [9,10]. Our case report at the time of publication demonstrated overall satisfactory outcomes for the patient in terms of completeness of tumor removal and final cosmetic appearance.

## Case presentation

A 39-year-old Afro-Caribbean female, originating from and residing in a tropical island close to the Equator, noticed a pigmented lesion on the skin of the right ala of the nose in January 2023. Initially, it was very small and almost indistinguishable in size and color from the surrounding normal skin. In February of the same year, it was 8 mm in diameter. It also became more prominently pigmented, especially after a day of exposure to the sun at the beach. She presented with concern after several months with what she presumed to be a sunburn that failed to resolve. She did not have any pain or serosanguineous discharge from the lesion at any time. She also did not have any history suggestive of sunburns or actinic keratosis, nor any family history of skin cancers.

**Figure 1:**
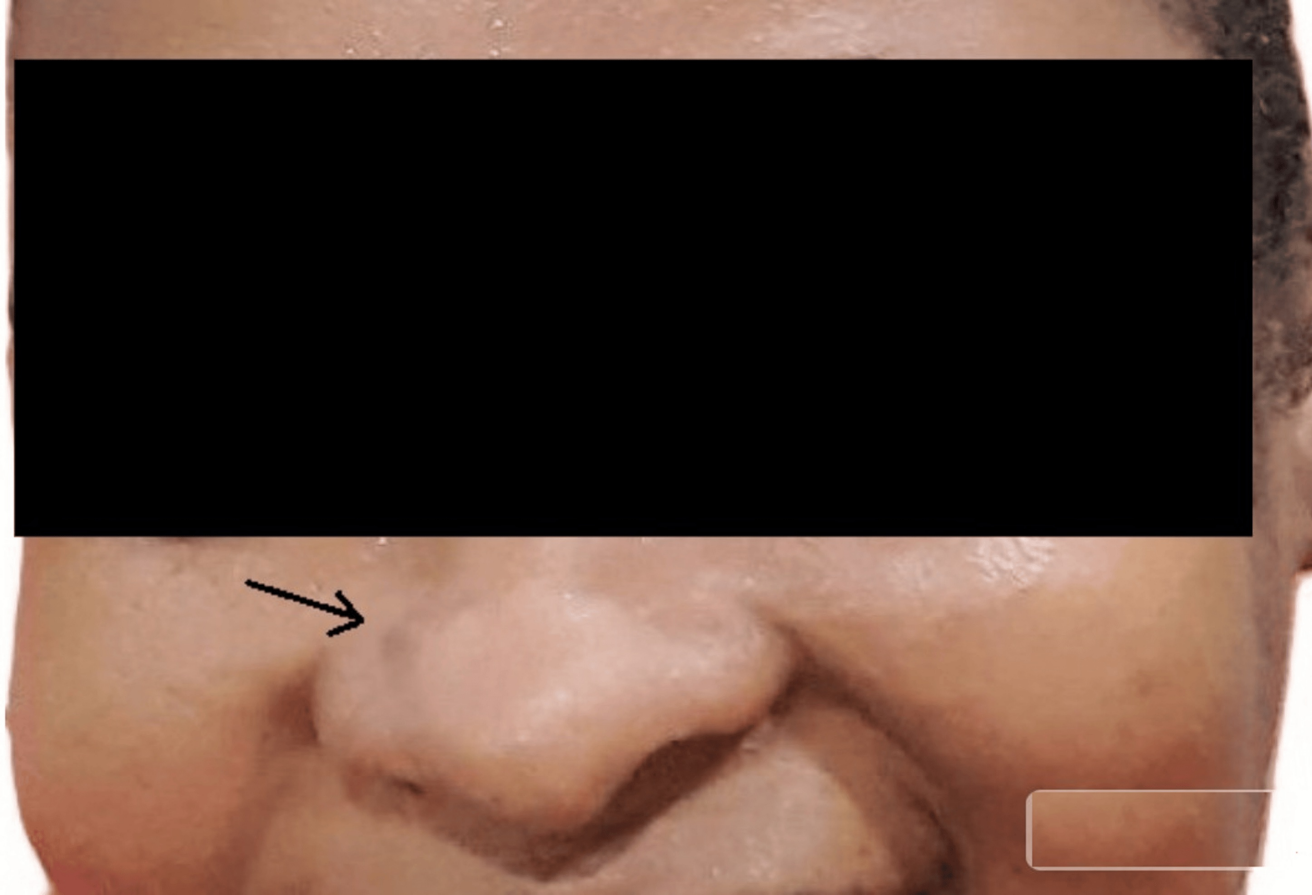
Local finding on the right ala of the nose in January 2023 The lesion is denoted with a black arrow.

**Figure 2:**
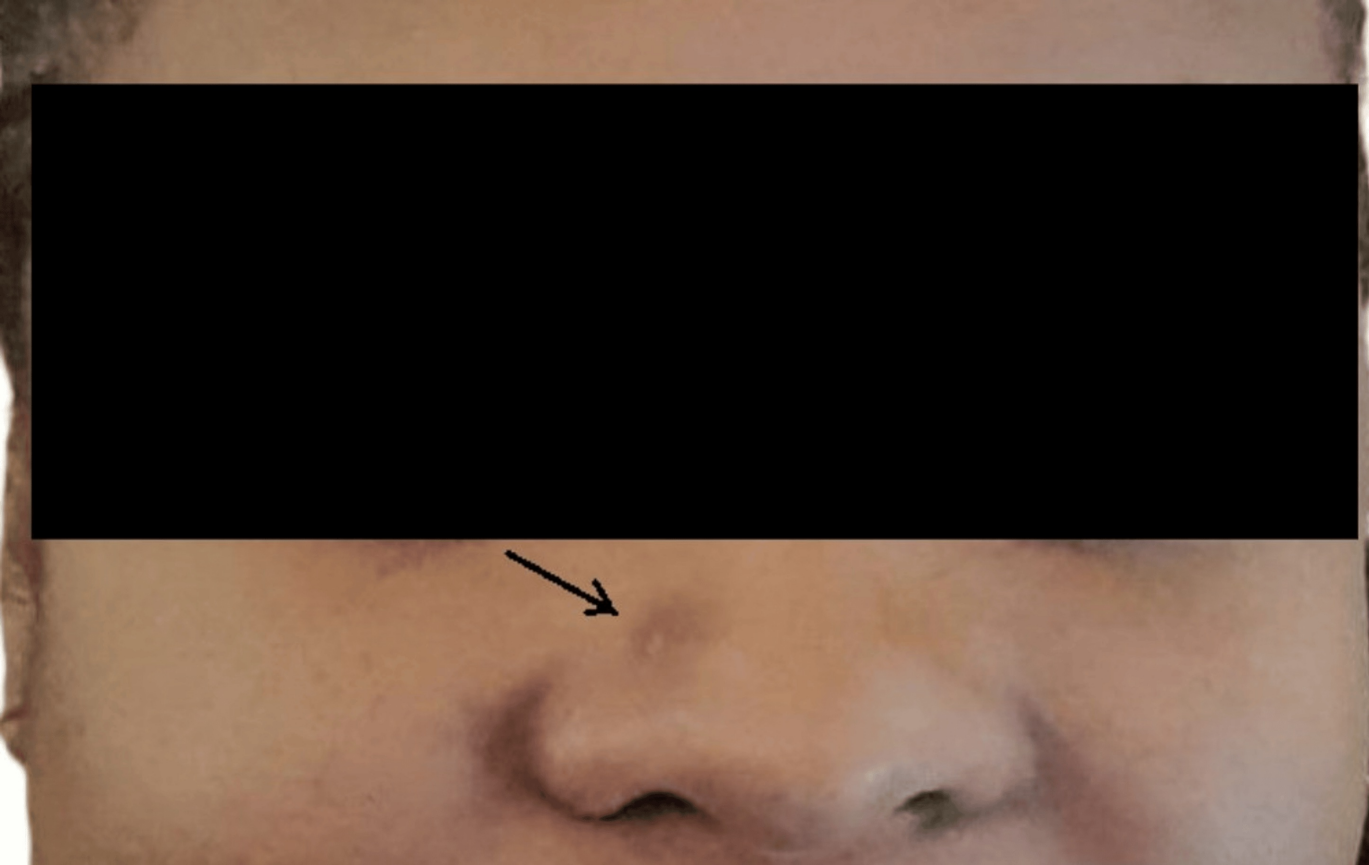
Local findings on the right side of the nose in August 2023 The lesion is denoted with a black arrow. The progression of the lesion over eight months, compared with the previous image (Figure 1), is evident.

Physical examination revealed an otherwise fit and healthy female, appropriate for age and ethnicity, with Fitzpatrick type V skin. Local examination in July 2023 revealed a 12 x 14 mm hyperpigmented lesion in the right nasal wing, with irregular and elevated borders and a central depression. There was no loco-regional lymphadenopathy. The local findings recorded in January 2023 and eventually in August 2023 are shown in Figures 1, 2 to highlight the temporal changes of the lesion over eight months.

**Figure 3:**
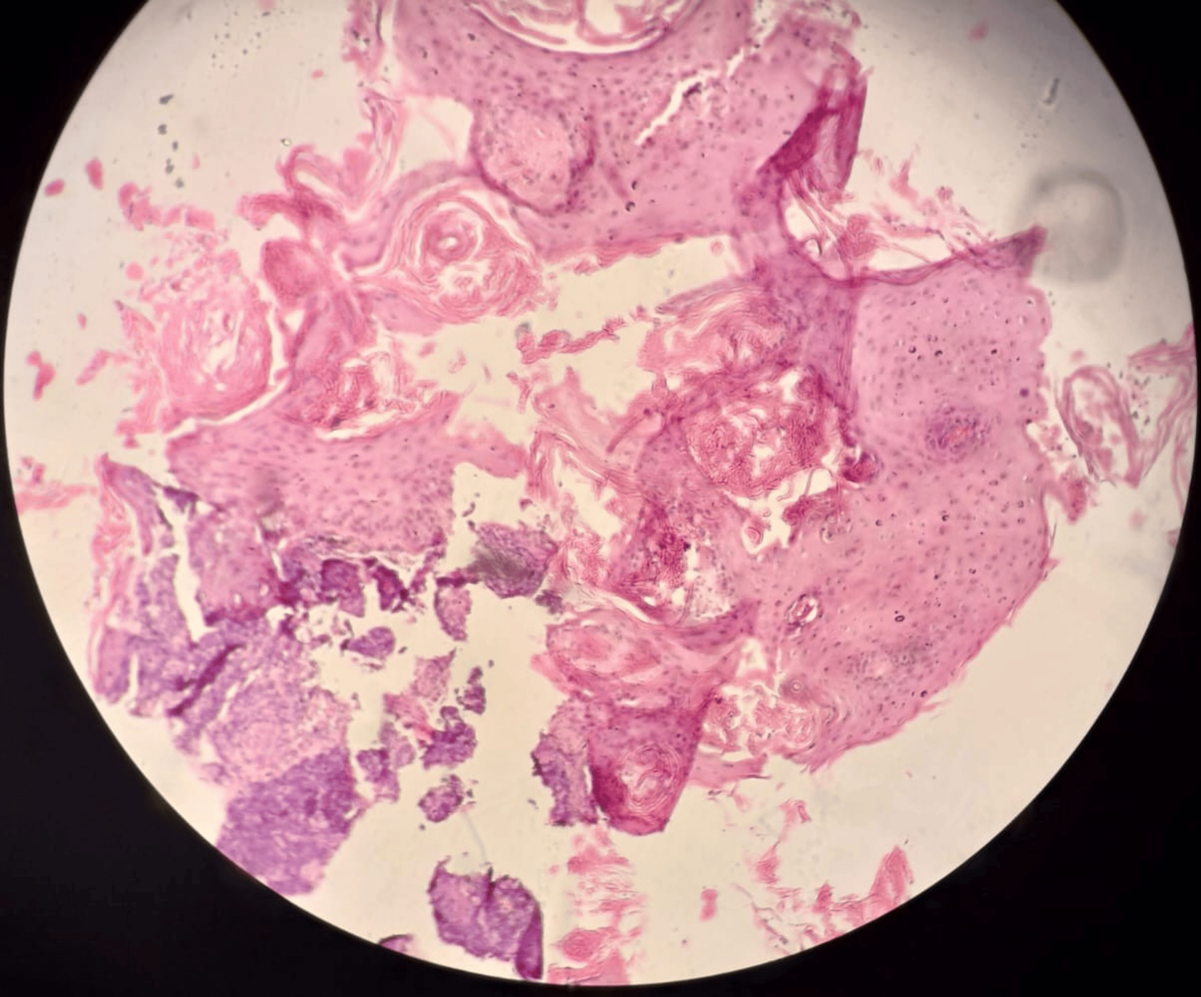
Photomicrograph of HPE of a biopsy specimen from the lesion (x200 magnification) The image demonstrates keratin-producing proliferation of atypical keratinocytes arising from the epidermis, with invasion into the underlying dermis. Eosinophilic concentric keratin pearls are also visible. Hematoxylin and eosin stain was used. HPE: Histopathological examination.

**Figure 4:**
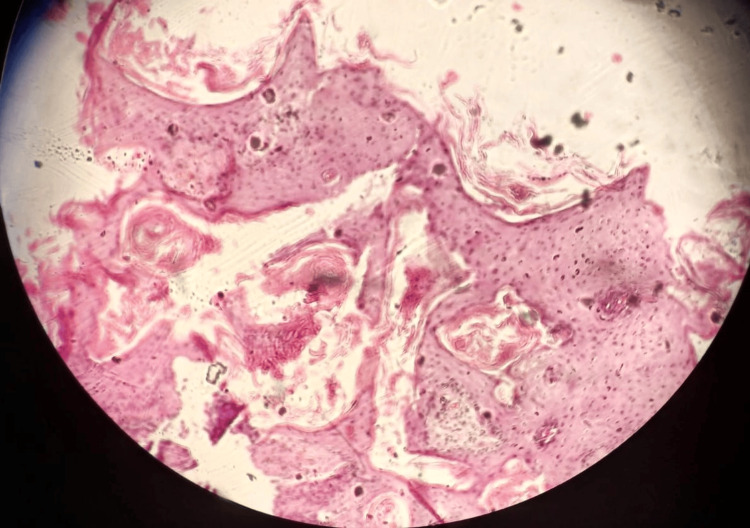
Photomicrograph of another HPE of a biopsy specimen from the same lesion (x200 magnification) Numerous mitotic figures, pleomorphism, and atypical forms are evident. Keratin pearl formations are also noticed. Hematoxylin and eosin stain was used. HPE: Histopathological examination.

The current description was a non-healing lesion of the right lateral nasal pyramid, progressively increasing in size, with a central depression. With a provisional clinical diagnosis of BCC, the ear-nose-throat (ENT) surgeon initially managing the patient performed an institutional biopsy under local anesthesia (LA). This was reported by the pathologist in April 2024 as keratinizing SCC. The histopathological examination (HPE) report showed nests and islands of atypical keratinocytes, dyskeratosis (premature and abnormal keratinization), invasive growth into stroma (dermis), hyperchromasia, and a high nuclear-to-cytoplasmic (N-C) ratio. There were several prominent eosinophilic kerato-hyalin pearls (concentric keratinization), indicating moderate to well-differentiation. All these features are illustrated in the photomicrographic images in Figures 3, 4.

Given these findings, the patient was referred to an oncologist in June 2024. Hematology, blood chemistry, and serology reports were within normal limits (WNL). CT scan of the head and neck did not reveal any metastatic lesions or other significant abnormalities. Based on the histopathological diagnosis, it was decided to perform a curative resection of the lesion, followed by reconstruction. However, due to a lack of resources on-island for cosmetic and reconstructive surgery of the face, the patient was further referred to an offshore center for definitive management in July 2024.

By September 2024, the preoperative size of the lesion was 1.1 x 2.0 cm. It was a large, keratotic, violaceous, sclerotic plaque on the right nasal lateral wall. From the plastic surgical standpoint, it was ascribed to area-H of the face. This is typically the "mask area" of the face, including the central face, eyelids, inner/outer canthi, eyebrows, nose, chin, lips, ears, periauricular skin, and temple. The etiology, diagnosis, and prognosis of the skin cancer were discussed with the patient. Various treatment options were reviewed, including but not limited to electrodessication and curettage, excision, radiation treatment, frozen sections, and Mohs surgery. Finally, Mohs surgery was decided upon, with secondary wound closure by flap and/or graft. The patient’s consent for the same was secured. The final indication for Mohs surgery was based on the anatomical location of the lesion, its propensity to recur, the type of tumor, its size, and its ill-defined borders.

In November 2024, prior to taking the first layer (A-layer) of Mohs surgery, given the slightly atypical clinical appearance of the lesion after the biopsy, a small shave sample was taken as a positive control before removing the A-layer. Stat frozen biopsy confirmed the diagnosis of well-differentiated invasive SCC (Figures 3, 4).

The patient was prepared in the usual manner, preparatory to excision of the A-layer of MMS. Anesthesia was obtained with local lignocaine infiltration. The clinically apparent tumor was debulked with a curette. A 1-2 mm rim of tissue was marked circumferentially around the defect. The area thus outlined was excised deep to the subcutaneous tissue. Hemostasis was achieved with electrocautery. The specimen was oriented, subdivided into two sections, chroma-coded with hematoxylin and eosin, and submitted for horizontal frozen sections. Upon review of the horizontal frozen sections for the A-layer, no residual tumor was identified.

**Figure 5:**
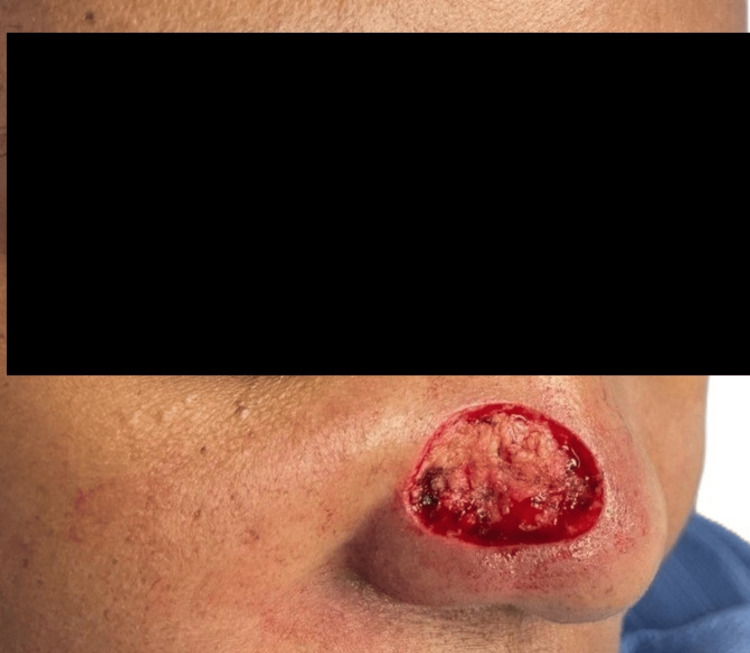
Surgical defect after Mohs micrographic surgery on a primary tumor from the right nasal pyramid Image showing the location of the original tumor as recorded on November 4, 2024, along with the size and location of the surgical defect.

The postoperative defect size was 2.1 x 1.8 cm (Figure 5). Given the large size (extending from the right ala to supra-tip to the right nasal sidewall, but not quite crossing midline), lack of redundant surrounding tissue (lack of solar elastosis/wrinkles/furrows), coupled with a relatively young age, and Fitzpatrick skin type V, other repair methods (melo-labial interpolation/melo-labial (cheek-lip) transposition/forehead flap) were considered to be inadequate options. Therefore, it was decided to allow the defect to granulate by secondary intention for two to three weeks, with a tentative plan for delayed FTSG.

**Figure 6:**
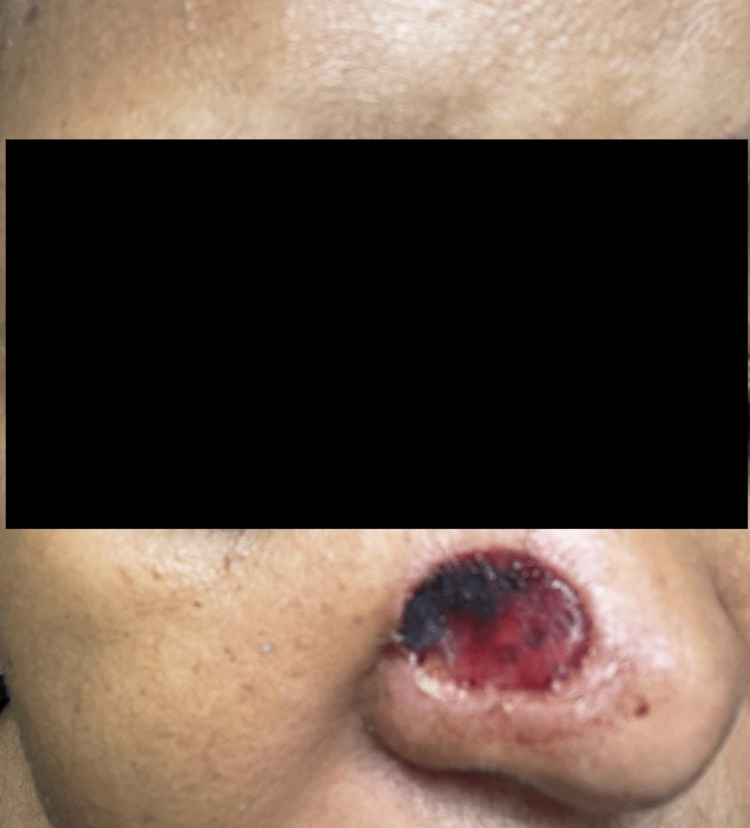
48 hours after Mohs micrographic surgery of the lesion on the right ala of the nose Image showing the clean margins and granulation in the wound depth.

**Figure 7:**
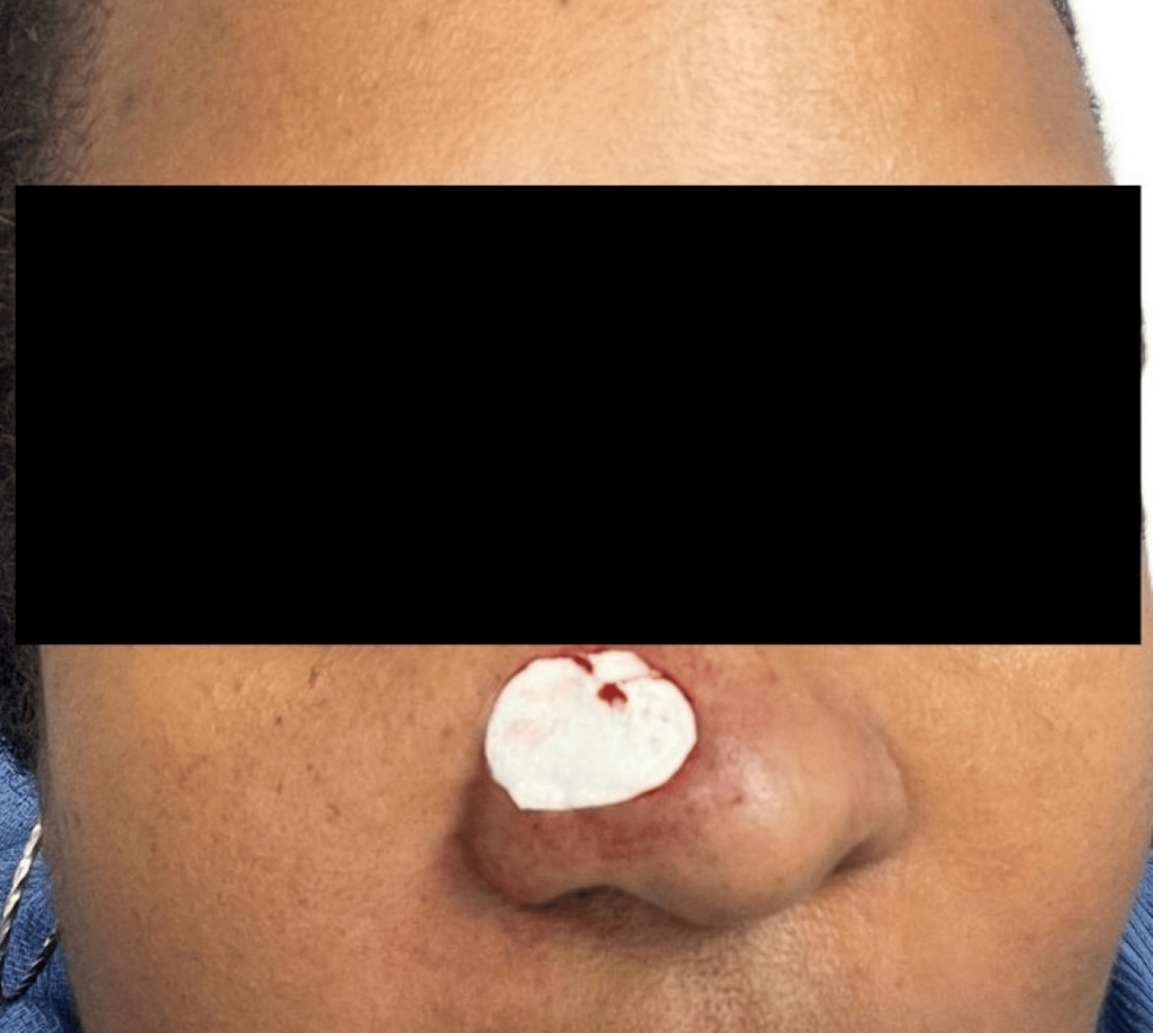
Alginate dressing in place over the wound defect following Mohs micrographic surgery of the nasal lesion Alginate facilitates wound healing. Further details regarding the use of alginate dressings in this case are described in the main text.

**Figure 8:**
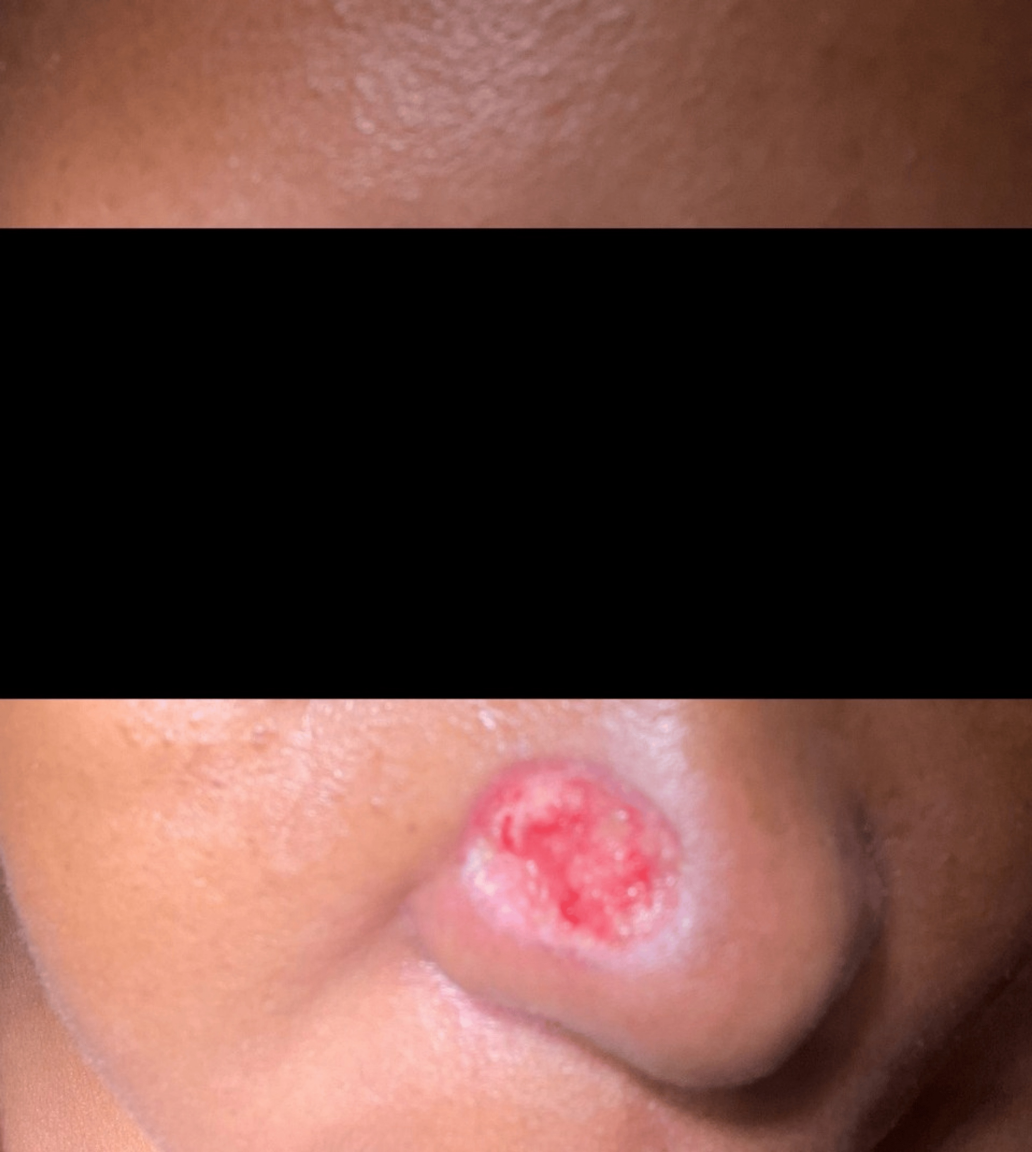
11 days postoperative after Mohs excision surgery of the nasal lesion Image showing pink, healthy, flat granulation tissue and the beginning of neo-epithelialization at the wound edges.

For the next two weeks, the patient underwent daily wound cleansing with hydrogen peroxide (H_2_O_2_), followed by alginate and antiseptic dressings. The wound showed healthy granulation with no signs of infection or undesirable exudates (Figures 6-8).

Given the size of the wound, the patient’s relatively young age, and Fitzpatrick skin type V, it was decided that FTSG would most likely offer the best combination of functional, esthetic, and expedient repair while minimizing the risk of contracture or alar rim elevation.

**Figure 9:**
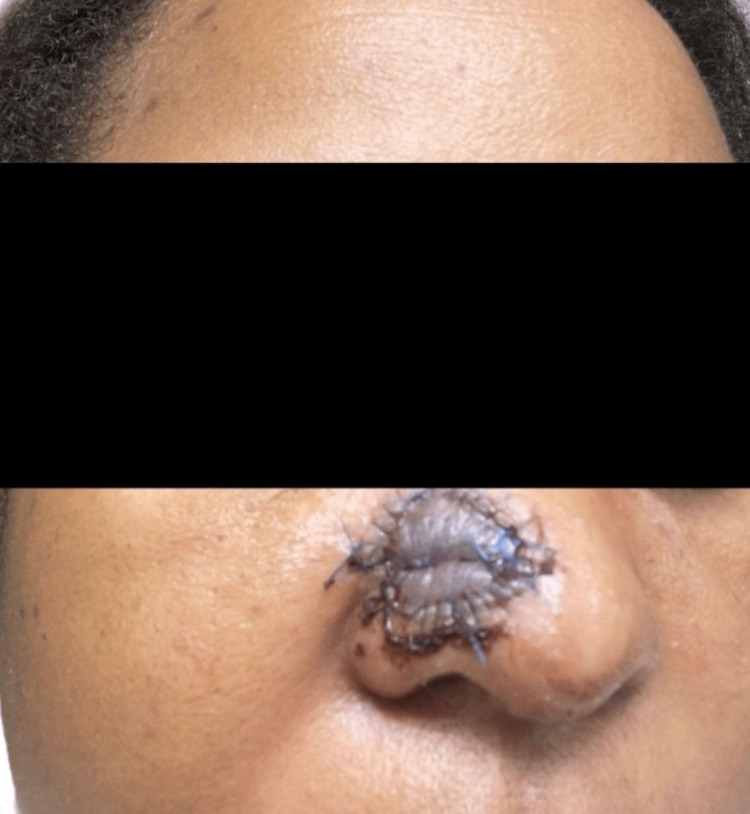
72 hours after secondary FTSG of the wound defect on the nose following Mohs excision of the lesion Image showing the circumferential and tacking blue Prolene sutures. FTSG: Full-thickness skin grafting.

**Figure 10:**
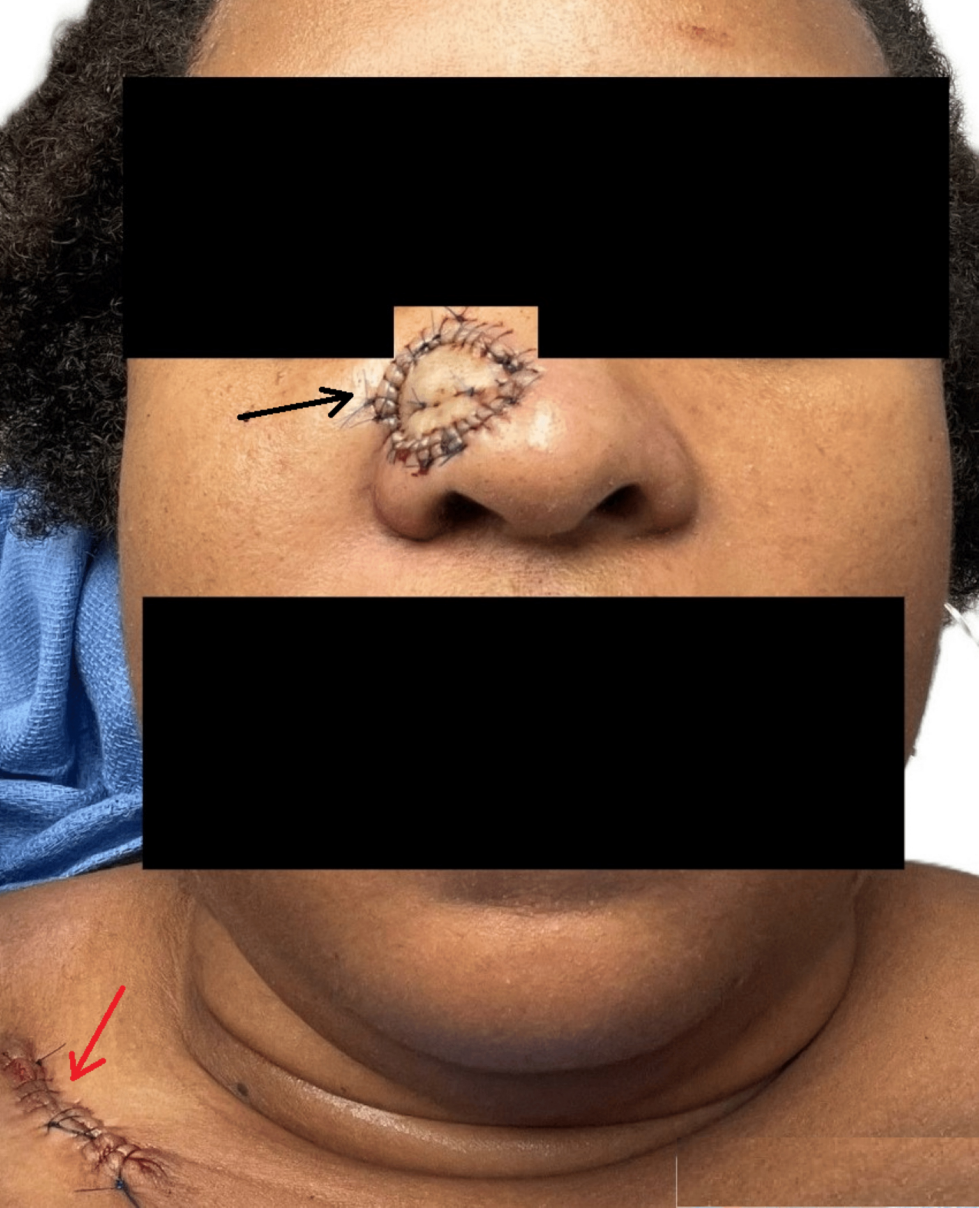
14 days after FTSG on the nasal surgical defect The donor area in the right clavicular region is also visible. Final size: right nasal graft recipient area (black arrow), 2.2 × 2.0 cm; right clavicular donor site (red arrow), 5.0 cm (linear). FTSG: Full-thickness skin grafting.

Two weeks after Mohs micrographic excision, the granulating wound was closed with FTSG taken from the right clavicular region. Optimal skin match was determined to be from the right clavicular region. The donor area was marked, prepped, and anesthetized. Full thickness of skin was excised through a linear incision down to the subcutaneous plane. Local undermining was performed, and hemostasis was obtained with electrocautery. The incision was closed with 3-0 Vicryl and 4-0 Prolene sutures in a layered fashion. The FTSG was shaped to match the nasal defect and defatted. LA was infiltrated, and hemostasis was obtained at the recipient site. The graft was sutured into place with running and interrupted 5-0 Prolene sutures. Tacking sutures were utilized to further secure the graft in place (Figures 9, 10).

FTSG graft sutures were removed on December 3, 2024. The graft had taken on to the entire recipient area on the right ala of the nose with 100% success. The patient was relieved that the tumor was removed and reported satisfaction with the overall result.

**Figure 11:**
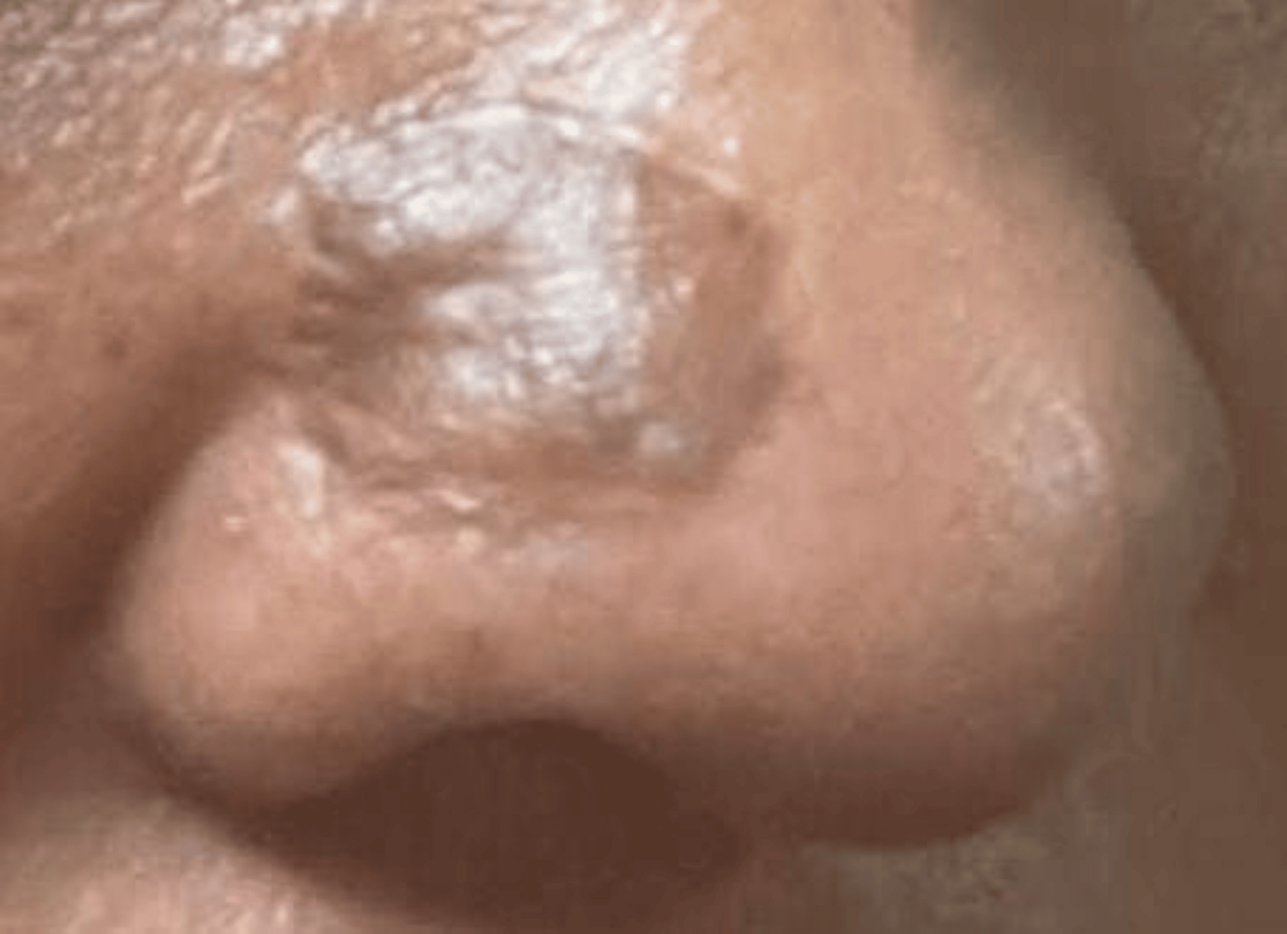
Appearance of the grafted area over the nose after 25 days of FTSG The image shows post-graft hyperpigmentation and slight pincushioning. The graft take rate was 100%. FTSG: Full-thickness skin grafting.

Slight hyperpigmentation and pincushioning (bumpy, pitted elevations) of the graft were noted. Pincushioning of the graft was treated with one sitting of intralesional steroid (ILS) injections, coupled with general topical measures, with considerable improvement in overall graft appearance. Plans for further sessions of ILS were made over the next few months (Figure 11).

**Figure 12:**
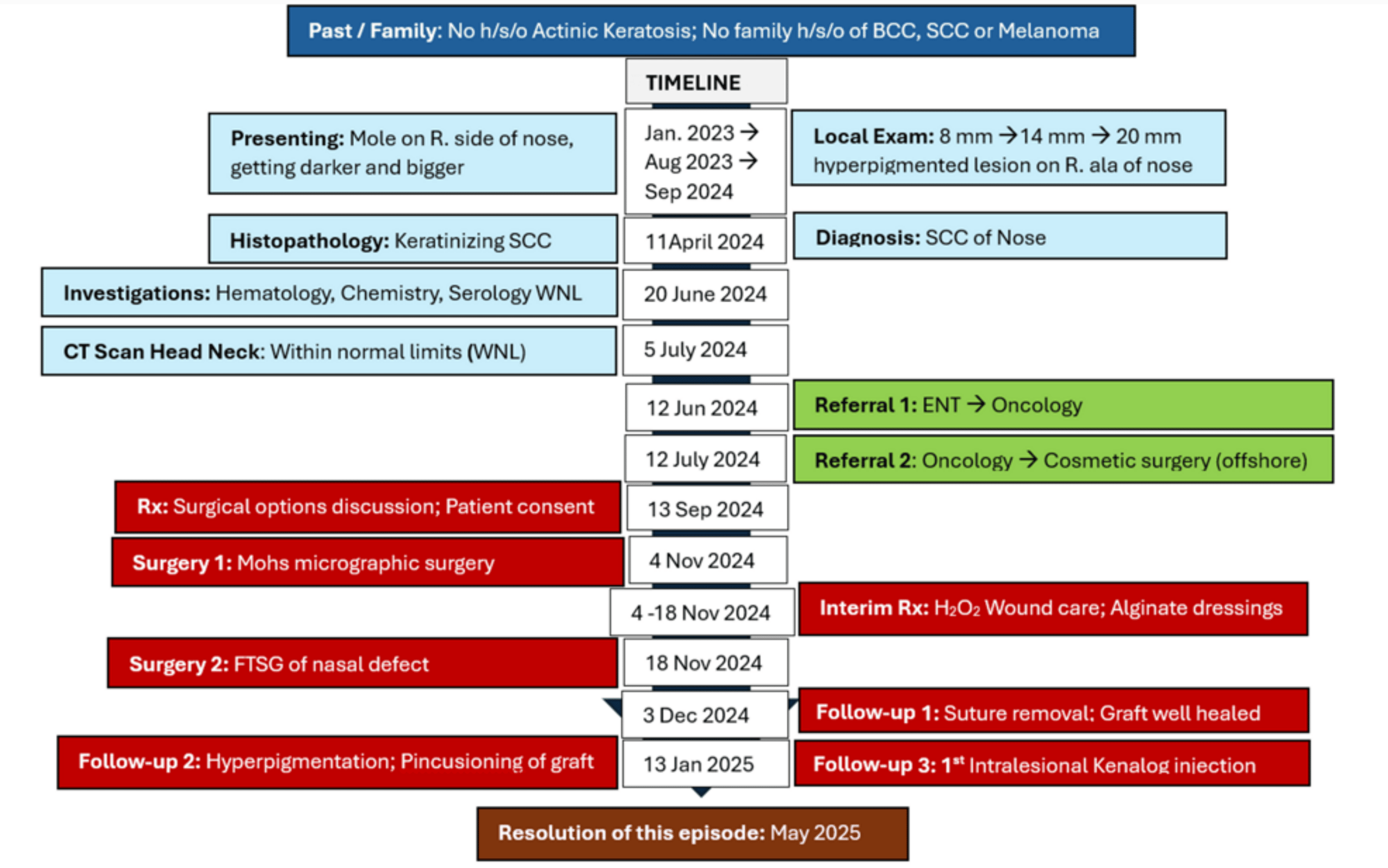
Timeline of case report SCC: Squamous cell carcinoma; BCC: Basal cell carcinoma; CT: Computerized tomogram; ENT: Ear, nose, throat; H_2_O_2_: Hydrogen peroxide; FTSG: Full-thickness skin graft.

The timeline of the case report from its inception in January 2023 to its conclusion in May 2025 is illustrated in the following temporal infographic (Figure 12).

## Discussion

BCC versus SCC

While our preoperative clinical diagnosis was BCC, two subsequent HPE reports of biopsy specimens from the same lesion proved it to be invasive, well-differentiated keratinizing SCC. Our preliminary diagnosis of BCC was based on certain epidemiological and demographic considerations. BCC is the most common of skin cancers worldwide [3-5]. Chronic high exposure to solar UV rays is the most important risk factor for BCC [6-8]. Our patient originated from the tropical islands and resided there all her life, with her frequent outdoor lifestyle. These islands close to the Equator are prone to high solar UV radiation. BCC tends to be diagnosed at a younger age than SCC. BCC is also significantly more common in younger age groups (around 40 years of age) compared to SCC [3-5]. Our patient presented with her current condition when she was 39 years old. Nodular BCC is the most common type and classically presents as a pink dome-shaped papule [3]. The initial appearance of the lesion in our patient was a dark brown, small nodular elevation (Figures 1, 2). Approximately 80%-90% of all BCC cases commonly occur on sun-exposed areas of the body, especially the head and neck. This is typically described as the "mask area" or H-zone of the face, which includes the central face, eyelids, nose, ears, and periorbital regions. Within the head and neck, specific common locations include the nose, which is often cited as the single most common site for BCC on the face. These areas are also considered high-risk for more aggressive tumor behavior and recurrence. SCC tends to be more aggressive than BCC, with a higher rate of loco-regional recurrence after inadequate and/or delayed excision. Therefore, a high index of suspicion, coupled with early, rather than late intervention, is advisable [3-5,8].

With these overlapping etiological, epidemiological, and demographic characteristics, SCC and BCC are very close in the differential diagnosis of skin cancers. The reported slow and progressive increase in size of the lesion in our patient over the months, with increased pigmentation after each sun exposure, was in favor of a putative diagnosis of BCC [3-5,8].

Fitzpatrick skin types versus UV radiation versus skin cancers

Dark-skinned people are classified in the higher end of Fitzpatrick skin type classification (types V and VI), as opposed to light-skinned people who are grouped in the lower end of the same classification (types I and II). Our patient was categorized as Fitzpatrick skin type V. This classification is based on the potential of the skin to tan or burn on exposure to solar UV radiation. Dark skins (types V and VI) rarely burn but tan easily and darkly. Light skins (types I and II) behave in the reverse way [6].

Most of the dermatologic effects of sunlight are caused by UV radiation, which is divided into three wavelength bands: UVA (320-400 nm), UVB (280-320 nm), and UVC (100-280 nm). Because of the stratospheric ozone (O_3_) layer's filtration of the radiation, only UVA and UVB reach the Earth’s surface. Sunburn-producing rays are typically of wavelengths < 320 nm, which is effectively UVB, since UVC hardly reaches the earth’s surface [7]. Adverse effects of UV exposure include acute sunburn and several chronic changes. Latter includes skin thickening, wrinkling, actinic (solar) keratosis, and cancer [7]. Therefore, it behooves us to ask for and look for these preexisting lesions when presented with a patient with suspected skin cancer.

BCC is the most common non-melanoma skin cancer, while SCC is the second most common. Both have distinct risk factors, though both often appear on sun-exposed areas like the face and hands. Both BCC and SCC primarily affect people with fair skin (Fitzpatrick skin types I and II) who burn easily. Thus, fair skin is a major risk factor for both. BCC tends to be linked to overall UV dose. Cumulative, long-term sun exposure plus intense, intermittent exposure (sunburns) tend to produce BCC more often. SCC is also strongly tied to intense sunburns and often arises from sun-damaged skin. Frequent, cumulative sun exposure, often from outdoor or recreational work, can lead to SCC. The latter sometimes develop from precancerous lesions like actinic keratoses. Both can occur in any skin color, though often presenting differently in darker skin. People with darker skin (Fitzpatrick types V and VI) can also get these cancers, often on less sun-exposed areas like legs, and may be diagnosed later. As age advances, the incidence becomes equal, and in some locations, SCC even becomes more prevalent than BCC. This is because of cumulative sun exposure with age, which is considered the single most important risk factor for both BCC and SCC [7,8].

Mohs micrographic surgery

Mohs surgery is named after its pioneer, Frederick E. Mohs, who performed the procedure in the 1930s while still a medical student. Initially, it was called Mohs "chemosurgery" because it involved fixation of the excised tissue, containing the tumor, with a zinc chloride paste, followed by microscopic examination 24 hours later for residual tumor [9]. Since then, Mohs surgery has evolved into a same-day MMS.

In classical MMS, first, the tumor is outlined. The tissue layer is precisely oriented by placing superficial etch marks with a scalpel (according to the cardinal points of a compass) around the tissue layer and corresponding in-situ skin. Under LA, any visible tumor is debulked with a scalpel. Thereafter, a thin margin of tissue is removed circumferentially around and deep to the debulked tumor defect at a beveled angle of 45 degrees, which facilitates tissue processing in the next step. The removed tissue layer is cut into halves or quadrants and marked with colored dyes (chroma-coded) to facilitate precise mapping of the tumor. The tissue is then pressed flat, so the epidermal edge occupies the same geometric plane as the deep layer. The tissue is then processed on a horizontal plane so that 100% of the peripheral and deep margins can be examined on the same tissue section under the microscope. This is in contrast to the traditional vertical tissue processing, which examines only about 1% of the tumor margin. If a residual tumor is identified under the microscope, then the Mohs map is re-marked, and the corresponding in-situ tissue is precisely removed again only in the portion that was found to still have the residual tumor. This process is repeated until the excised specimen is histologically negative. This sequential process allows 100% of the excised tissue margins to be examined within 15-30 minutes on the same day. It ensures complete tumor removal with maximum conservation of healthy tissue. Mohs surgery is particularly suitable for areas of the body in the H-area: central face, eyelids/canthi, eyebrows, nose, lips, chin, ear, and periauricular area [9,10].

In our patient, the lesion was on the nose, which is a cosmetically sensitive and geometrically uneven area. The skin of the ala of the nose is thin, and the subcutis contains thin muscle fibers of alar nasalis that are almost indistinguishable from the surrounding tissue. This is followed by the U-shaped mobile hyaline alar cartilage, covered with perichondrium on both surfaces. The interior of the ala of the nose (vestibule of the nose) is again lined by skin, containing hair follicles. Given this architecture of the ala of the nose, MMS provided the ideal solution for complete removal of the lesion while achieving its two principal objectives, namely 100% tumor removal and maximum normal tissue-sparing with minimal disfigurement.

A variety of techniques are used to close the defect resulting from tumor removal. These include primary closure, flaps, grafts, and allowing healing by secondary intention. Flaps for nasal defects may include melolabial (cheek-lip) interpolation flaps, forehead flaps, and melolabial transposition flaps [11]. The technique described in our patient involved allowing the wound to granulate for two weeks while pursuing H_2_O_2_ cleansing and alginate dressing, followed by FTSG.

Healing by secondary intention - alginate dressings

Alginate dressings work on surgical wounds by absorbing exudate and forming a soft gel with a moist environment. They provide hemostasis via ion exchange between calcium in dressings and sodium ions in the blood, thereby activating the clotting cascade and helping to stop bleeding. The gel structure ensures the dressing does not stick to the wound, allowing for painless removal and preventing damage to delicate new tissue. They conform to irregular wound shapes and deep cavities, ensuring contact with the entire wound bed and facilitating wound healing from within and from the margins by modulating macrophage activity. They stimulate natural angiogenesis (neovascularization) and tissue regeneration. Thus, they are great for surgical sites with drainage and allow for easy, atraumatic removal, making them ideal for deep, irregular, draining, and bleeding wounds, as amply demonstrated in our patient [12].

Pincushioning of the skin graft

Pincushioning of a skin graft or flap is a cosmetic complication, especially on the nose, where the reconstructed skin tends to be raised above the surrounding skin and looks bumpy like a "pincushion." It creates an aesthetically unpleasant, lumpy appearance, which may be due to scar contracture, tissue bunching, excess fat, and lymphatic or venous obstruction. It happens as healing scars pull inward, forcing underlying tissue from the flap or graft to bulge outward. It can be prevented by wide undermining of the surrounding skin prior to grafting and making straight rather than curved incisions. If preventive techniques do not work, it may require defatting of excess adipose tissue, dermabrasion, laser therapy, surgical revisions like Z-plasty, or ILS injections, like intralesional Kenalog in our case, to flatten the area [13].

Limitations of the study

Even though the patient reported overall satisfaction with the results and relief that the entire tumor was removed successfully, this study, being a single case report, suffers from the drawback of limited generalizability. Moreover, a greater long-term follow-up of the patient is required to determine the 5- and 10-year tumor-free status and overall success of MMS in this case.

## Conclusions

BCC and SCC, while respectively the two most common non-melanoma skin cancers worldwide, are also close differentials of each other, often constituting a diagnostic conundrum. A high index of suspicion is required in susceptible individuals, including those with certain Fitzpatrick skin types, those residing in regions with a high UV index and habitual UV exposure, and those with family and/or past history of skin cancers. This should be followed by early histopathological diagnosis, which can lead to expeditious management decisions, especially if it entails offshore referral. The judicious application of tissue-sparing techniques, like MMS, ensures a high rate of tumor-free survival, maximum normal tissue-sparing, and minimal cosmetic disfigurement. A larger number of cases with longer follow-ups will be more useful in terms of generalizability and overall outcome assessment.

## References

[1] Falto-Aizpurua L, Griffith R, Simmons B (2015). The history of skin cancer. J Am Acad Dermatol.

[2] Kasper M, Jaks V, Hohl D, Toftgård R (2012). Basal cell carcinoma — molecular biology and potential new therapies. J Clin Invest.

[3] Puckett Y, Steele RB (2025). Basal Cell Carcinoma. StatPearls Publishing.

[4] Bichakjian C, Armstrong A, Baum C (2026). Basal cell carcinoma: clinical presentation and management. Basal Cell Carcinoma: Clinical Presentation and Management. PCMed Project Dermatology.

[5] Bartoš V, Kullová M (2018). Non-melanoma skin cancer - a clinicopathological study of patients with basal cell carcinoma and squamous cell carcinoma. Klin Onkol.

[6] MSD Manual (2026). Fitzpatrick skin type classification. Fitzpatrick Skin Type Classification.

[7] Benedetti J (2026). Overview of effects of sunlight. https://www.msdmanuals.com/professional/dermatologic-disorders/reactions-to-sunlight/overview-of-effects-of-sunlight.

[8] (2026). American Academy of Dermatology Association. Types of skin cancer. https://www.aad.org/public/diseases/skin-cancer/types/common.

[9] (2026). American college of Mohs surgery. About Mohs Surgery.

[10] Prickett KA, Ramsey ML (2025). Mohs Micrographic Surgery. https://www.ncbi.nlm.nih.gov/books/NBK441833/.

[11] Cerci FB, Kubo E (2020). Nasal reconstruction after Mohs micrographic surgery: analysis of 208 cases. Surg Cosmet Dermatol.

[12] Aderibigbe BA, Buyana B (2018). Alginate in wound dressings. Pharmaceutics.

[13] Amici JM (2014). [Early hypertrophic scar after surgery on the nasal region: value of long-acting corticosteroid injections]. Ann Dermatol Venereol.

